# Developmental Social Environment Imprints Female Preference for Male Song in Mice

**DOI:** 10.1371/journal.pone.0087186

**Published:** 2014-02-05

**Authors:** Akari Asaba, Shota Okabe, Miho Nagasawa, Masahiro Kato, Nobuyoshi Koshida, Takuya Osakada, Kazutaka Mogi, Takefumi Kikusui

**Affiliations:** 1 Companion Animal Research, School of Veterinary Medicine, Azabu University, Sagamihara, Kanagawa, Japan; 2 Kato Acoustics Consulting Office, Yokohama, Kanagawa, Japan; 3 Division of Electronic and Information Engineering, Tokyo University of Agriculture and Technology, Tokyo, Japan; 4 Department of Applied Biological Chemistry, Graduate School of Agricultural and Life Sciences, The University of Tokyo, Tokyo, Japan; National Institute of Allergy and Infectious Diseases, United States of America

## Abstract

**Background:**

Sexual imprinting is important for kin recognition and for promoting outbreeding, and has been a driving force for evolution; however, little is known about sexual imprinting by auditory cues in mammals. Male mice emit song-like ultrasonic vocalizations that possess strain-specific characteristics.

**Objectives:**

In this study, we asked whether female mice imprint and prefer specific characteristics in male songs.

**Methods and Findings:**

We used the two-choice test to determine the song preference of female C57BL/6 and BALB/c mice. By assessing the time engaged in searching behavior towards songs played back to females, we found that female mice displayed an innate preference for the songs of males from different strains. Moreover, this song preference was regulated by female reproductive status and by male sexual cues such as the pheromone ESP1. Finally, we revealed that this preference was reversed by cross-fostering and disappeared under fatherless conditions, indicating that the behavior was learned by exposure to the father's song.

**Conclusions:**

Our results suggest that female mice can discriminate among male song characteristics and prefer songs of mice from strains that are different from their parents, and that these preferences are based on their early social experiences. This is the first study in mammals to demonstrate that male songs contribute to kin recognition and mate choice by females, thus helping to avoid inbreeding and to facilitate offspring heterozygosity.

## Introduction

Sexual imprinting is important for kin recognition and for promoting outbreeding, and has been a driving force for evolution. During sexual imprinting, females develop species-specific strategies for selecting mating partners. When given a choice, female mice in mating or preference assays prefer males whose major histocompatibility complexes (MHC) are different from their own [1]. Mice are thought to be capable of kin recognition that is largely mediated by olfactory cues. When female mice were cross-fostered and raised by the fostering parents, they showed a reverse preference to MHC odor [2]. This preference is determined by early learning experiences, so called “sexual imprinting.” However, mating behaviors are mediated by multiple sensory cues; whether, and how, different sensory cues may contribute to mate preference remains unclear.

**Figure 1:**
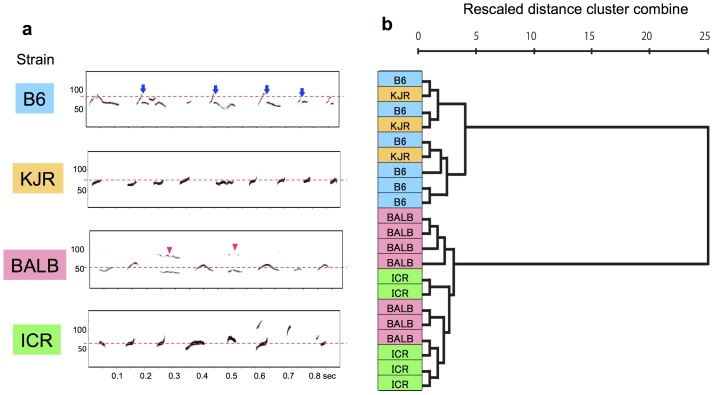
Strain-specific characteristics of male mouse songs. (**a**) Representative spectrogram of the songs from B6, KJR, BALB, and ICR. Arrows and arrowheads indicate the “Jump” (B6) and “Harmonic” waveforms (BALB), respectively. The dotted lines indicate the average peak frequency of each song (B6, 79 kHz; KJR, 75 kHz; BALB, 55 kHz; ICR, 66 kHz). (**b**) Cluster analysis using the following parameters: mean peak frequency of syllables, interval between syllables, duration of syllables, and percentage composition, for each waveform category among B6, BALB, ICR, and KJR male individuals (B6, *n* = 6; BALB, *n* = 7; ICR, *n* = 5; KJR, *n* = 3). There were 2 major song groups: BALB and ICR, and B6 and KJR.

Male and female animals exchange multiple sensory signals, not just olfactory cues, during interaction. Male mice emit ultrasonic vocalizations, the features of which are similar to the calls of songbirds [3], when they encounter females or female urinary pheromones. We, and others, have reported that the characteristics of adolescent mice and male mouse songs differ across strains and the characteristics of the songs are immutable in cross-fostering experiments [4]–[6]. The song of C57/BL6 (B6) males showed a higher peak syllable frequency, shorter intervals between syllables, and more “Jump” syllables, whereas those of BALB/cA (BALB) males included more “Harmonics” syllables (see Fig. 1a). We recently demonstrated that the structure of these mouse song sequences is under strong genetic control [5], which is in contrast with song production in songbirds [7]. Several studies have demonstrated that female mice move towards male songs [8]–[10], suggesting that the songs of male mice are attractive to female mice. However, whether female mice prefer specific traits in male songs remains to be elucidated.

Here, by simultaneously presenting male songs from 2 discrete ultrasonic speakers to female mice, we examined whether female song recognition also has a genetic component. We found that females can recognize and prefer strain-specific song characteristics that differ from those of their parents. Moreover, multisensory information is important for inducing adaptive behavior; for example, maternal retrieving behavior is regulated by multisensory information from pup olfactory and auditory cues [11]. Olfactory cues from males are important in stimulating female mating preference and sexual receptive behavior in mice [2], [12]. Therefore, in the present study, we investigated whether male odor additionally or synergistically stimulated female responses to male songs.

Finally, we investigated whether female preference for male songs is innately regulated by genetic control, or shaped by social experience during the developmental period as has been shown in songbirds [13]–[15]. We revealed that this preference was reversed by cross-fostering and disappeared under fatherless conditions, indicating that this behavior was learned by exposure to the father's presence. This is the first study to show that the developmental social environment shapes female preference for male songs in mice.

## Results

### Strain-specific character of male songs

We recorded and analyzed songs from 4 strains of male mice (Movie S1): B6, BALB, KJR, and ICR; the representative spectrograms of the songs are shown in Fig. 1a. Each syllable was identified as 1 of 9 distinct categories (“Upward”, “Downward”, “Flat”, “Short”, “Chevron”, “Complex”, “One jump”, “More jumps,” and “Harmonics”) according to previous studies [5], [6]. The syllable categories showed a significant effect of strain in MANOVA (Roy's Largest Root = 19.074, *F* = 28.611, *p*<0.0001, Fig. S1a). The B6 song contained more “One jump” and “More jump” syllables (*p*<0.001, *p*<0.001, respectively), whereas the BALB song contained more “Harmonics” (*p*<0.001) compared to the other strains. In addition, the KJR song contained more “Flat” (*p*<0.001), and the ICR song had more “Downward” syllables (*p*<0.01). The songs produced by males of these 4 strains were different from one another in song profile: peak frequency (ANOVA, *F*_3,17_ = 27.493, *p*<0.0001, Fig. S1b), syllable interval (ANOVA, *F*_3,17_ = 12.550, *p*<0.001, Fig. S1c), and syllable duration (ANOVA, *F*_3,17_ = 7.136, *p*<0.0001, Fig. S1d), demonstrating that some characteristics of male songs differed across strains. We performed cluster analysis to elucidate similarities and dissimilarities among the features of each strain's song, using the following parameters calculated for each individual mouse: average peak frequency, interval between syllables, average syllable duration, and probability of occurrence of each syllable type [5]. As a result, ICR and BALB songs were categorized in one group, while KJR and B6 songs ware categorized in a separate group (Fig. 1b), indicating both similarities and dissimilarities among male songs.

### Female mice prefer the songs of different strains when stimulated by male sexual chemosignals

**Figure 2:**
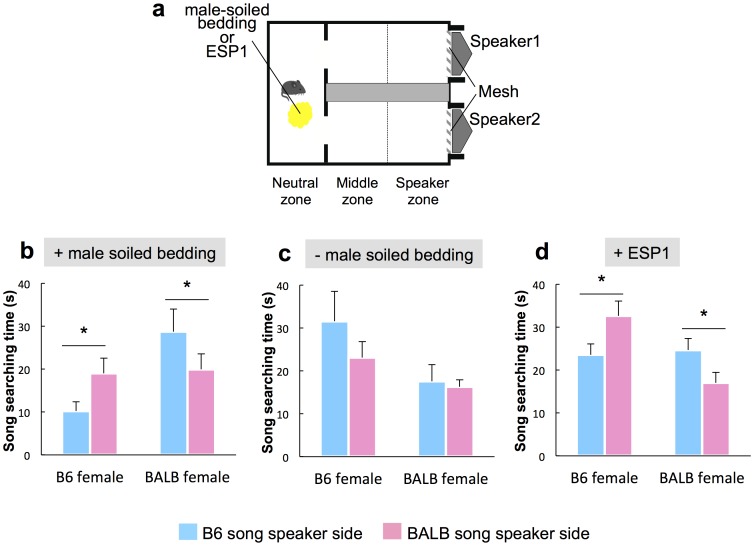
Female mice prefer songs of males from different strains. (**a**) Schematic of the apparatus used for the playback experiment. (**b**) B6 (*n* = 6) and BALB (*n* = 10) females in diestrus exposed to male-soiled bedding before testing showed longer-duration search times for other strains' songs. (**c**) Duration of time searching during diestrus in B6 (*n* = 5) and BALB (*n* = 7) females in the absence of male odor before testing. (**d**) B6 (*n* = 11) and BALB (*n* = 13) females in diestrus exposed to male pheromone ESP1 before testing showed longer duration search times for other strains' songs. Values represent means+standard error. Asterisks indicate significant differences p<0.05.

We investigated the preference of B6 and BALB females in each estrous cycle for the songs of their own or different strains of male mice, in the presence of male sexual chemical cues to simulate internal and external natural conditions. B6 and BALB songs, which contained the most distinctly different characteristics among the 4 strains, were played back simultaneously to female mice; the time engaged in searching behavior towards a mesh placed in front of the speaker was assessed as a behavioral preference (Fig. 2a). When female subjects were exposed to male-soiled bedding (a mixture of B6 and BALB as chemosignals) before testing, B6 females in diestrus spent significantly more time searching towards BALB male songs than towards B6 male songs (Generalized linear model (GLM), Waldχ^2^ = 7.366, *p* = 0.007; Wilcoxon signed-ranks tests, *p* = 0.0464; Fig. 2b). In contrast, BALB females in diestrus spent more time searching towards B6 songs (*p* = 0.0469; Fig. 2b). However, these preferences were not observed in other estrous stages (Fig. S2a, b), when females were not exposed to male-soiled bedding (Fig. 2c and Fig. S2c, d), or when females were exposed to female-soiled bedding (Fig. S3). These results suggested that both B6 and BALB females showed preferences for songs produced by males of other strains in the diestrus stage, when stimulated by male sexual cues (Movie S2).

It is likely that pheromone cues that trigger sexual responses are responsible for the observed behaviors. However, male-soiled bedding contains many chemosignals that are components of urine, feces, skin cells, and hair, which confound interpretation of this observation. The exocrine gland-secreting peptide 1 (ESP1), as we previously reported, has been shown to induce female sexual receptivity [12]. We therefore used ESP1 as a male cue to elucidate the effects of male sex signals. Following exposure to ESP1, females from both strains of mice significantly preferred songs produced by males of a different strain (GLM, Waldχ^2^ = 12.37, *p* = 0.006; Wilcoxon signed-ranks tests, B6, *p* = 0.0409 and BALB, *p* = 0.0392; Fig. 2d), indicating that male sexual cues enhance female preference for songs.

### Song preference is based on dissimilarity of song character

**Figure 3:**
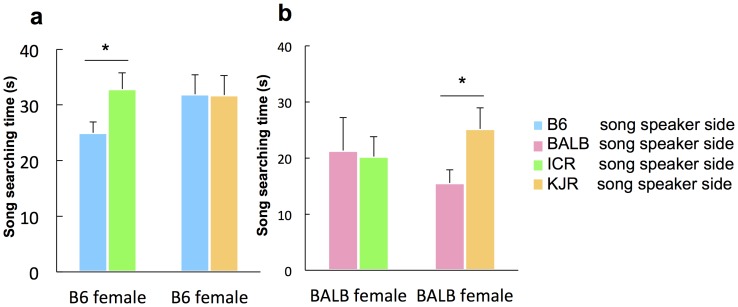
Female song searching response to playback with KJR and ICR strain male song. (**a**) When B6 and ICR male songs were presented, ESP1-treated B6 females showed longer search times for ICR songs than for B6 songs (*n* = 13), whereas there was no difference in search time when B6 and KJR male songs were presented (*n* = 12). (**b**) When BALB and KJR male songs were presented, ESP1-treated BALB females showed longer search times for KJR songs than for BALB songs (*n* = 12), whereas there was no difference when BALB and ICR male songs were presented (*n* = 11). Values represent means+standard error. Asterisks indicate significant differences p<0.05.

Playback sound files used in the experiments shown in Fig. 2 and in Figs. S2 and Figs. S3 were recorded from a particular mouse from each strain. To confirm the generality of the preference for individual songs produced by males of the same strain, we played back songs produced by different B6 and BALB males (Fig. S4). Even in this experiment, females showed a preference for songs produced by males from a different strain (GLM, Waldχ^2^ = 9.64, *p* = 0.002; Wilcoxon signed-ranks tests, B6, *p* = 0.028; BALB, *p* = 0.043). We also performed tests with songs produced by males of the other strains, ICR and KJR. The two-choice test revealed that B6 females preferred ICR songs over B6 songs (*p* = 0.033, Fig. 3a, Left), but not KJR songs (Fig. 3a, Right). BALB females preferred KJR songs over BALB songs (*p* = 0.041, Fig. 3b, Right), but not ICR songs (Fig. 3b, Left), demonstrating that females preferred songs that were dissimilar to those produced by males of their own strain.

### Song preference was sharpened by the father's presence during development

**Figure 4:**
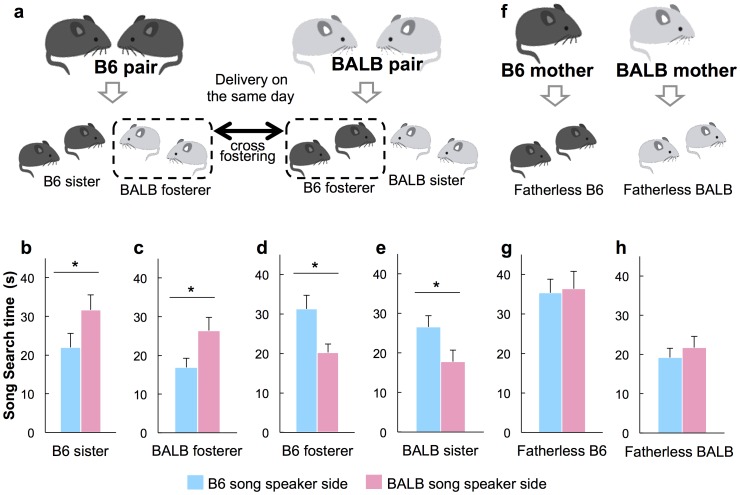
Cross-fostering reversed, and fatherless rearing diminished, female song preference. (**a**) Experimental scheme of the cross-fostering paradigm. Part of the litter (B6-fosterer and BALB-fosterer) was reciprocally cross-fostered to parents of the other strain of mice. (**b–e**) B6 sister (*n* = 10) and BALB-fosterer (*n* = 15) showed longer search times for BALB song. In contrast, B6-fosterer (*n* = 16) and BALB sister (*n* = 14) treated with ESP1 prior to the experiment showed longer search times for B6 song. GLM with fosterer/sister and male song as factors revealed significant effects of the interaction. (**f**) Fatherless B6 and fatherless BALB ware raised by mothers only. (**g–h**) There was no significant difference in search time for B6 and BALB songs in fatherless B6 and BALB females. Values represent means+standard error. Asterisks indicate significant differences p<0.05.

To establish the relative contributions of genetic determination and environmental influence on development of song preference in mice, we studied the song preference of B6 females raised by BALB foster parents, and that of BALB females raised by B6 foster parents (B6 fosterers and BALB fosterers, Fig. 4a). B6 and BALB sisters exhibited behaviors similar to normal B6 and BALB females described above, respectively (GLM, Waldχ^2^ = 19.76, *p* = 0.0001; Wilcoxon signed-ranks tests, B6 sister, *p* = 0.0284, Fig. 4b; BALB sister, *p* = 0.0413, Fig. 4e). In contrast, BALB fosterers preferred BALB song and B6 fosterers preferred B6 song (BALB fosterer, *p* = 0.0413, Fig. 3c; B6 fosterer, *p* = 0.0409, Fig. 4d). In addition, song preference disappeared when females were raised in a fatherless condition (Fig. 4f–h). These results clearly demonstrated that female preference for male songs depends on auditory exposure to the father's presence during the pre-weaning period.

## Discussion

Here, we show for the first time that female mice can discriminate among the distinct characteristics of male songs and prefer the songs of mice of strains different to those of their parents. This preference occurs in the presence of male pheromones and, depends on estrous cycle. Moreover, we clearly demonstrate that song preference is acquired during early development through exposure to the presence of the father.

Preference for some sex traits might be acquired during evolution, if females receive benefits such as reproductive success [16]. Our recent study revealed that the sequences of male mouse songs are under strong genetic control [5]. These findings suggest that the preference for songs of males from a different strain contributes to disassortative mating, which is an important “mate choice” strategy for avoiding inbreeding and facilitating heterozygosity of offspring.

The finding that wild derived female mice showed greater preference for songs produced by unfamiliar than by familiar males [9] strongly supports our results. Moreover, we found that female song preference was reversed by cross-fostering, and did not appear if females were raised under fatherless conditions, indicating that female mice learn the characteristics for mate preference from the father during the pre-weaning period. It is possible that female pups were exposed to litter mates' ultrasound calls, but the characteristics of these calls were different from those of adult males [4], [17]. Adult male-type song production initiates at an age of approximately 55 days; therefore, exposure to the father's song may be more important for imprinting female preference during the pre-weaning period. One possibility of father's song exposure to female pups was the time that the mother was in postpartum estrus after delivery and the father and mother mice were sexually active [18]. We attempted to monitor ultrasound vocalizations in pairs of B6 and BALB mice for 3 weeks after pup delivery to obtain preliminary data. Despite technical difficulties in accurately defining the subject producing the ultrasonic vocalization, these results confirmed that parents and pups communicate during the first 3 weeks of pups' lives. We found complex father song-like spectrograms after the day of pup delivery (data not shown), and we sometimes found two overlapping types of spectrograms (e.g., “complex” ultrasonic syllables and squeaks) that may reflect warbling (data not shown). Even if 1- to 2-day-old offspring are unable to hear their father's song, they later have the opportunity in the form of exposure to intrafamilial vocal communications that constructs future female song preference. However, future studies are needed in order to have a better understanding of intrafamilial vocal communications.

To investigate female preference for male song in strains other than B6 and BALB, we conducted play-back tests using songs produced by ICR and KJR males. Clustering analysis (Fig. 1b) showed that ICR and BALB songs were categorized in one group, while KJR and B6 songs ware categorized in a separate group. Thus, there was a certain level of similarity was observed among the strains (relative similarity between ICR and BALB songs and between KJR and B6 songs). B6 females showed no preference for KJR songs (Fig. 3), suggesting that these females cannot discriminate between B6 and KJR songs. This is because of the similarity between the songs of these strains. Similarly, BALB females cannot discriminate between BALB and ICR songs. Interestingly, as shown by parametric analysis of song profiles (Fig. S1b), the peak frequencies of B6 and KJR songs are significantly different from those of BALB and KJR songs. Thus, it is possible that female song preference is based on song frequency, i.e., females prefer songs of mice from strains that produce different frequencies that those of their parents. An investigation of female preference for frequency-altered male songs may help to demonstrate this possibility.

We also found that song preference was dependent on phase of the estrous cycle. Social investigation towards an unfamiliar male was enhanced by moderate levels of estrogen [19], and the preference of BALB females for the urine of B6 males over that of BALB males was observed in the diestrus stage but not the estrus stage [20]. Previous studies demonstrated that estrous females showed more preference for masculine males (males with higher social rank or higher testosterone levels) than for those with lower social rank or castrated males [10], [21], [22]. In the present study, the number of songs emitted, which is controlled by testosterone [23], was equally standardized, and it was thought that the level of masculinity expressed in the songs was almost equal. Thus, female preference observed here may reflect kin recognition and avoidance of inbreeding rather than selection of more masculine mice. We hypothesize that females have two systems or strategies in mate choice: one is the preference for masculine males, which is observed clearly in the estrous phase; the other is avoidance of inbreeding, observed in the diestrous phase. Further research are needed to investigate diestrus female keep together with male singing songs preferred by the female and mate with that male in the estrus phase.

In addition to vocal communication, olfactory signals have been suggested to have significant effects on mate choice in mice. The preference revealed in this study was observed when females were exposed to male-soiled bedding or to ESP1, which enhance female sexual receptivity, before testing [12]. This result suggests that cross-modal information processing from both acoustic and olfactory signals may exist for mate preference in mice. Both auditory and odorant preference for mate choice were imprinted in female mice; this integrated sensory system may have plasticity that is dependent upon the social experience during the pre-weaning period. The neural mechanism for this processing is not clear, but this multimodal system can provide a novel basic model for cognitive neuroscience.

In conclusion, this study uncovers new possibilities for studying the biological significance of song variation, and the molecular and neural mechanisms of song perception in an established model genetic organism.

## Methods

### Animals

C57BL/6 (B6) and BALB/c (BALB) mice were originally obtained from Japan Clea Co. Ltd., and bred in our laboratory. Food and water were provided ad libitum, and animals were housed under a standard 12 L∶12 D cycle (lights on at 0600 h). An adult male and female mice of the same strain were pair-housed in a cage (17.5×24.5×12.5 cm). After weaning at 21 d after birth, the litters were housed in same-sex groups of 2–5 mice in a cage of the same dimensions as the breeding cage. All female mice were used in the experiment after they reached sexual maturity (7–35 wk old). To avoid litter effects, each experimental group was obtained from 1–2 females from the same litter, such that at least 4 to 5 litters were used in the same groups. Males used as singers were sexually mature (20–30 wk old) and experienced in mating and parenting behavior so that male social and sexual status was controlled. All experiments were conducted in accordance with the guidelines of “Policies Governing The Use of Live Vertebrate Animals” of Azabu University, and approved by The Ethical Committee for Vertebrate Experiments of Azabu University (ID#070418).

### Recording and creation of playback sounds

Ultrasound recording was performed using a condenser microphone (UltraSoundGate CM16/CMPA, Avisoft Bioacoustics, Berlin, Germany) as previously reported [5]. When recording songs from adult males, the microphone was placed beside a cage (12×20×11 cm) with 6-cm diameter holes and covered with a 0.5-cm wide mesh. Ultrasonic sounds were recorded for 3 min when a male mouse encountered a devocalized female. Females were devocalized by sectioning of the inferior laryngeal nerve according to previous report [24]. Playback sounds consisted of 20 s of ultrasounds representing the strain character from 2 individuals each of B6 (number of syllables, 133 and 162) and BALB (number of syllables, 108 and 166). Similarly, playback sounds of ICR (number of syllables, 106) and KJR (number of syllables, 133; kindly gifted by Dr. Koide of the National Institute of Genetics, Mishima, Japan) were prepared. Figure S5 shows the principal components in 6 playback songs used in each behavior test. These sounds were repeatedly generated during the tests. Before each playback experiment, we played the song and simultaneously observed the sound pressure levels in the experimental box by using the same microphone kept away from the speaker, and adjusted the pressure levels.

### Apparatus used for the two-choice test

The test cage was constructed using clear Plexiglas (42.5×27×25 cm), modified for assessing females' preference for 1 of 2 male songs reproduced through a nanocrystalline silicon thermo-acoustic emitter positioned at the end of each compartment (Fig. 2a). In our previous experiments, we confirmed that this speaker can produce a very accurate ultrasound from 20 to 150 kHz without a decline in the sound pressure levels [25]. The females could move into 1 of 2 sound compartments that were separated by an acoustic foam partition. Two speakers were placed at the opposite end of the cage through 6-cm diameter holes covered by 0.5-cm wide wire mesh, for reproducing male songs; 1 speaker was placed on each side of the acoustic partition. The sides and tops of the speakers were insulated with acoustic foam to ensure that playbacks were audible only in the corresponding cage compartment. To analyze female preference behavior, the compartments were further divided into 4 proximity zones: the “Mesh zone” (mice searching and in contact with the wire mesh), “Speaker zone” (an area 0–10 cm from the mesh), “Middle zone” (an area 10–20 cm from the mesh), and the “Neutral zone” (the undivided section of the test cage) (Fig. 2a). In the Neutral zone, females could hear reproduced songs equally from both speakers, confirmed by the sound intensity of each speaker. The test cage was placed in a sound-proof chamber during the experiment.

### Two-choice preference test protocol and statistical analyses

A female mouse was introduced into the Neutral zone, which was temporarily separated from the other compartments by 2 mobile partitions. After an acclimatization period, the mobile partitions were removed and the male songs of B6 and BALB mice were simultaneously reproduced for a 5-min period. The subject's behavior was analyzed during this period. Each experiment was recorded using a CCD camera connected to a video monitor, and the following factors were recorded: the number of entries into the sound compartment, the duration of stay in each speaker zone and middle zone, and the duration of searching time on the mesh. To present mouse chemical signal cues, male-soiled bedding or ESP1 was placed into the Neutral zone to increase the female's sexual arousal before starting the 5-min test. Female-soiled bedding was used as a control stimulus. The male- and female-soiled bedding was a mixture of 2 g each from adult B6 and BALB/c males and females respectively (3 mice per strain). Recombinant ESP1 (20 µg) in Tris buffer was transfused onto a piece of cotton (30 mg) and dried for 1 h before exposure, as previously described [12]. Each female was exposed to the soiled bedding for 15 min, or to ESP1 for 30 min, before initiation of the test.

### Estrus cycle and hormonal treatments

Vaginal smears were obtained to determine the phase of the estrous cycle 5–7 h before the trial. The smears were stained with Giemsa and examined under a light microscope at ×20 magnification. The stage of the estrus cycle was determined according to the ratio of cell types found in the smears, and was classified as pro-estrus, estrus, or metestrus and diestrus.

### Cross-fostering and fatherless condition

A male and female mouse of the same strain were pair-housed in a cage for breeding. Pregnant females were examined every 6–8 h for delivery. When pups were born at the same examined 6 to 8-h time period in both parental strains, a part of the litter was reciprocally cross-fostered to parents of the other mouse strain (B6-fosterer and BALB-fosterer). Control mice were handled in the same manner as fostered pups but were returned to their own parents (B6-sister and BALB-sister). When female mice were raised in fatherless conditions, a pregnant female was singly housed and monitored daily for delivery. All litters were left undisturbed until weaning postnatal day 21 (PD21). After PD21, female pups were housed with other females of the non-cross-fostered control (B6-sister or BALB-sister) of the different strain until the test day.

### Statistical analyses

Each behavioral factor was statistically compared between the 2 played-back sounds using a generalized linear model (GLM) with main factors, followed by Wilcoxon signed-ranks tests comparing the 2 sound compartments (SPSS statistical software, ver. 19). Some parameters were not normally distributed; thus only nonparametric tests were used. Female (except those represented in Fig. 3) were used only once. Half of the females in Fig. 3 were tested twice within an interval of >2 wk. There was no effect of repetition of the tests, and data from females used one and twice were combined.

## Supporting Information

Movie S1 **Singing male mouse.** A representative example of male courtship song emission. When a male mouse encountered a female, the male emitted a song-like ultrasonic vocalization during approach to the female. The recorded sounds were frequency-converted to 1/32 lower by audio generator software (Adobe Audition, version 2.0).
(MP4)

Movie S2 **Song preference.** A representative example of the preference test in a B6 and BALB female for B6 and BALB male songs.
(MP4)

Figure S1 **Parametric analysis of song profiles.** (**a**) Mean percentage composition for each syllable category in the 4 strains of mice. Percentages were the average probability calculated in each subject. (**b**) Mean peak frequency, (**c**) inter-syllable interval, and (**d**) duration of syllables in songs of each mouse strain. Different letters above bars represent significant differences at α = 0.05.
(TIFF)

Figure S2 **In the absence of male odor stimuli, or when in pro-estrous or estrus, females did not show a preference for songs of males of a different strain.** (**a**) Duration of time searching for songs during pro-estrus in B6 (*n* = 7) and BALB (*n* = 6) females exposed to male odor before testing. (**b**) Duration of time searching during estrus in B6 (*n* = 6) and BALB (*n* = 6) females exposed to male odor before testing. (**c**) Duration of time searching during pro-estrus in B6 (*n* = 5) and BALB (*n* = 5) females in the absence of male odor before testing. (**d**) Duration of time searching during estrus in B6 (*n* = 6) and BALB (*n* = 6) females in the absence of male odor before testing. There were no significant differences among these conditions.
(TIFF)

Figure S3 **In the presence of female odor stimuli, female mice did not show a preference for songs of males of a different strain.** Duration of time searching during diestrus in B6 (*n* = 5) and BALB (*n* = 7) females exposed to female odor for 15 min before testing. Female-soiled bedding contained a mixture of 2 g each from adult B6 and BALB/c females. No significant differences were observed.
(TIFF)

Figure S4 **Females showed preference for songs produced by different individuals of B6 and BALB males.** When songs were recorded from individual B6 and BALB males and played back to a female subject, B6 females (*n* = 6) showed longer search times for BALB song, and BALB females (*n* = 5) showed longer search times for B6 song. Asterisks indicate significant differences p<0.05.
(TIFF)

Figure S5 **Information about male songs used for each playback test.** (a) Principal components in 6 songs used for each playback test. (b) Mean percentage composition of each waveform category in the 6 playback songs.
(TIFF)
