# Supplementary figures and images for: Developmental Social Environment Imprints Female Preference for Male Song in Mice
Source: PLoS One. 2014 Feb 5;9(2):e87186. doi: 10.1371/journal.pone.0087186 (PMC3914833; doi:10.1371/journal.pone.0087186)

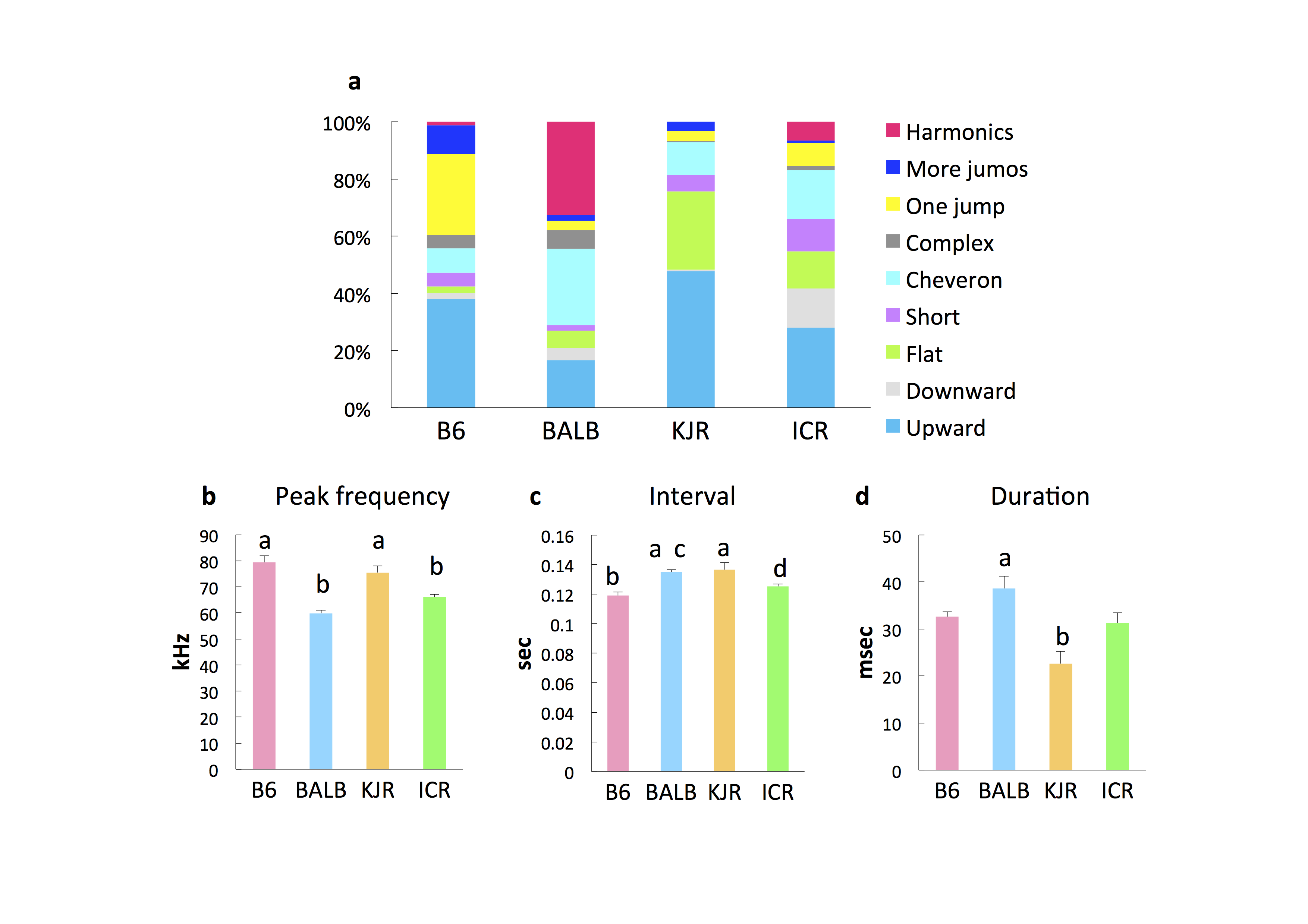

Supplement: Figure S1 — Parametric analysis of song profiles. (a) Mean percentage composition for each syllable category in the 4 strains of mice. Percentages were the average probability calculated in each subject. (b) Mean peak frequency, (c) inter-syllable interval, and (d) duration of syllables in songs of each mouse strain. Different letters above bars represent significant differences at α = 0.05. (TIFF) [file pone.0087186.s003.tif]

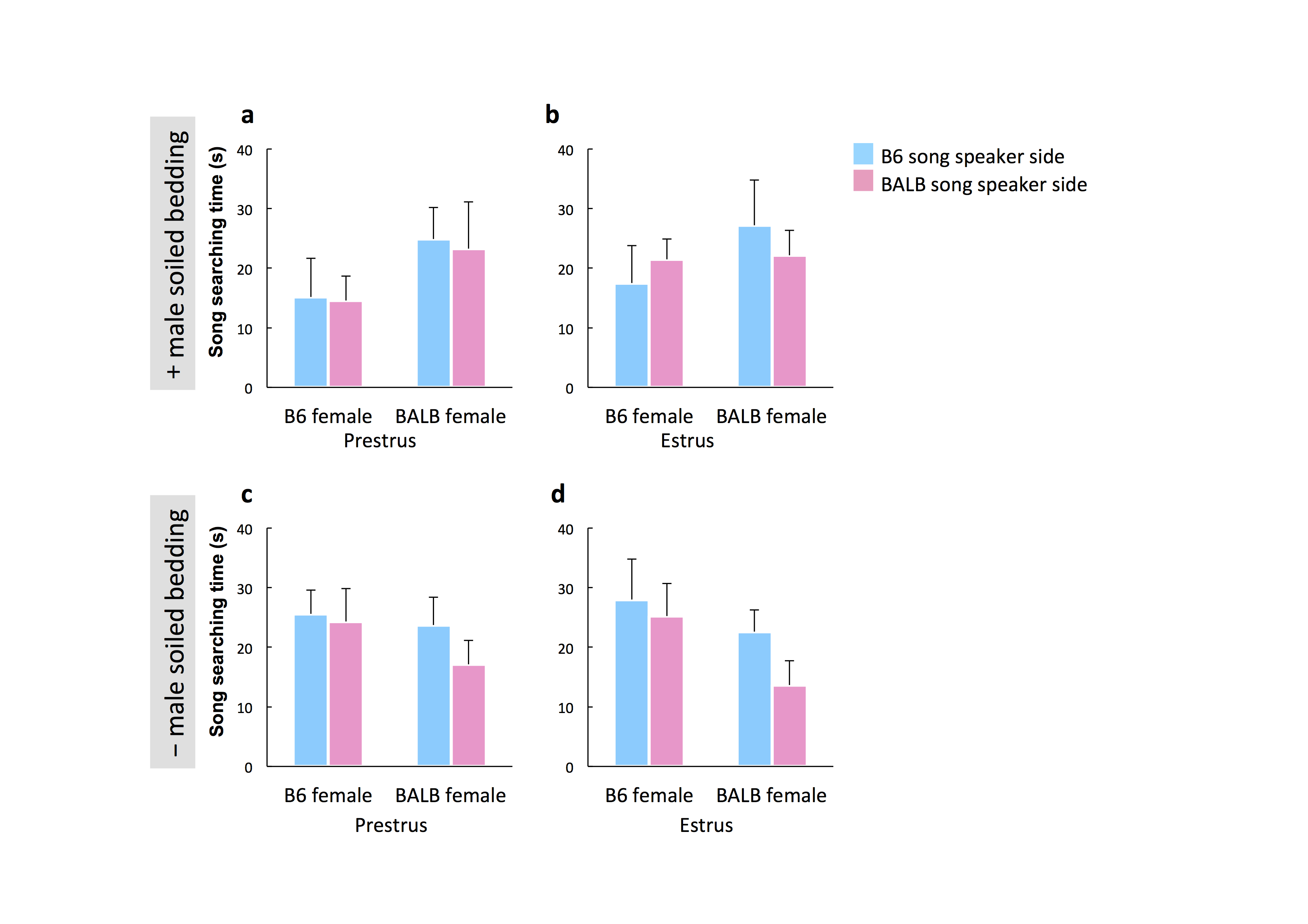

Supplement: Figure S2 — In the absence of male odor stimuli, or when in pro-estrous or estrus, females did not show a preference for songs of males of a different strain. (a) Duration of time searching for songs during pro-estrus in B6 (n = 7) and BALB (n = 6) females exposed to male odor before testing. (b) Duration of time searching during estrus in B6 (n = 6) and BALB (n = 6) females exposed to male odor before testing. (c) Duration of time searching during pro-estrus in B6 (n = 5) and BALB (n = 5) females in the absence of male odor before testing. (d) Duration of time searching during estrus in B6 (n = 6) and BALB (n = 6) females in the absence of male odor before testing. There were no significant differences among these conditions. (TIFF) [file pone.0087186.s004.tif]

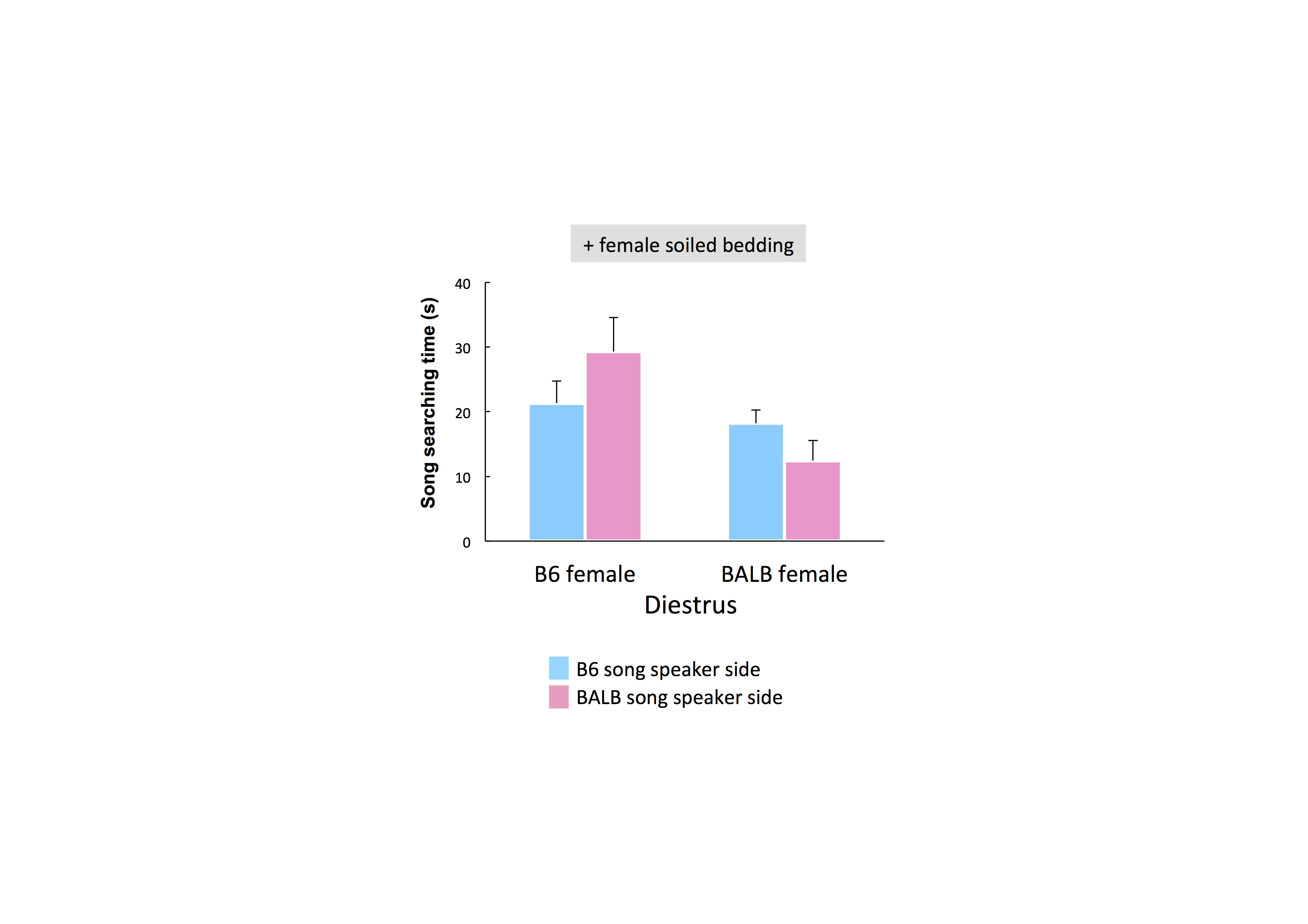

Supplement: Figure S3 — In the presence of female odor stimuli, female mice did not show a preference for songs of males of a different strain. Duration of time searching during diestrus in B6 (n = 5) and BALB (n = 7) females exposed to female odor for 15 min before testing. Female-soiled bedding contained a mixture of 2 g each from adult B6 and BALB/c females. No significant differences were observed. (TIFF) [file pone.0087186.s005.tif]

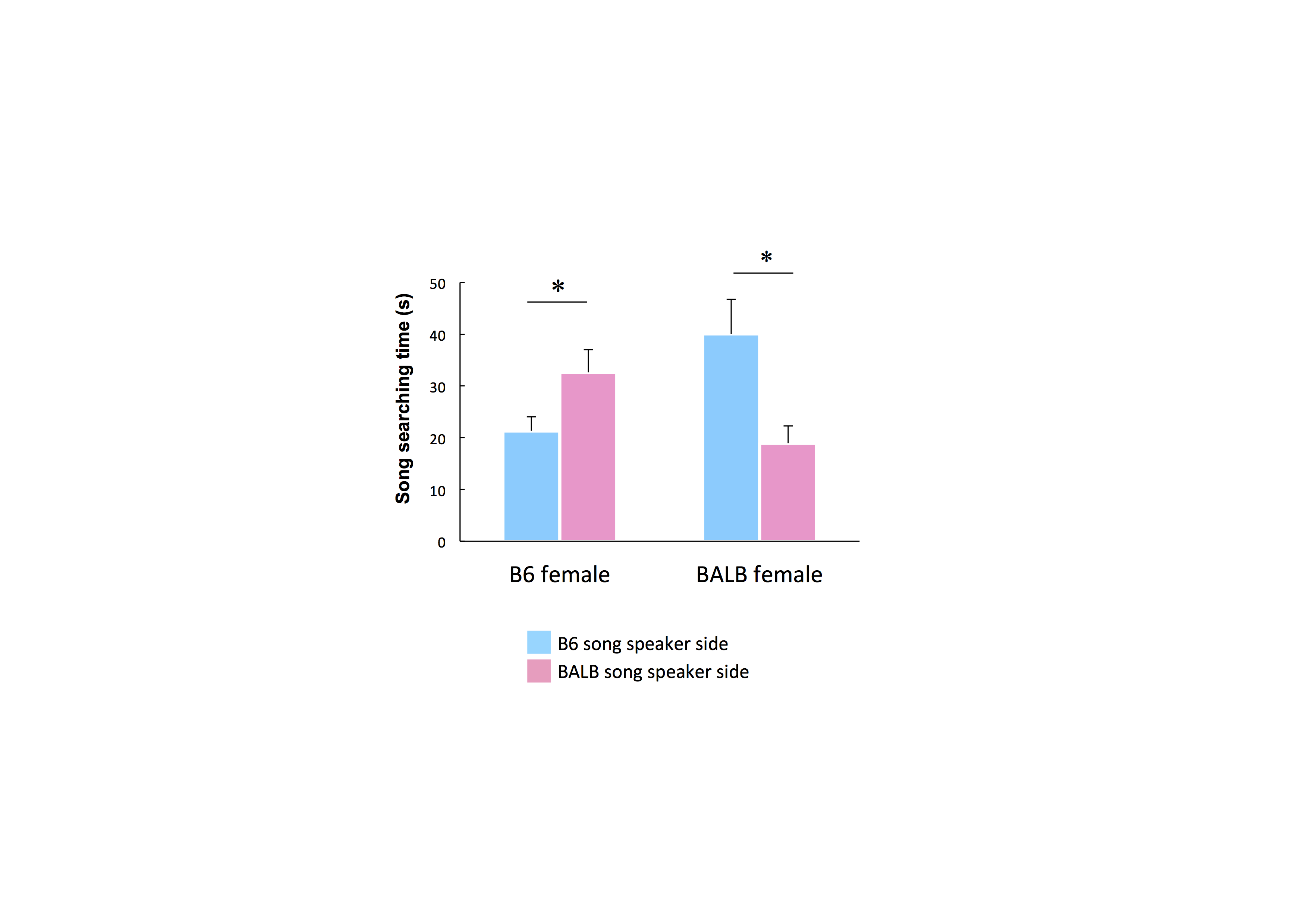

Supplement: Figure S4 — Females showed preference for songs produced by different individuals of B6 and BALB males. When songs were recorded from individual B6 and BALB males and played back to a female subject, B6 females (n = 6) showed longer search times for BALB song, and BALB females (n = 5) showed longer search times for B6 song. Asterisks indicate significant differences p<0.05. (TIFF) [file pone.0087186.s006.tif]

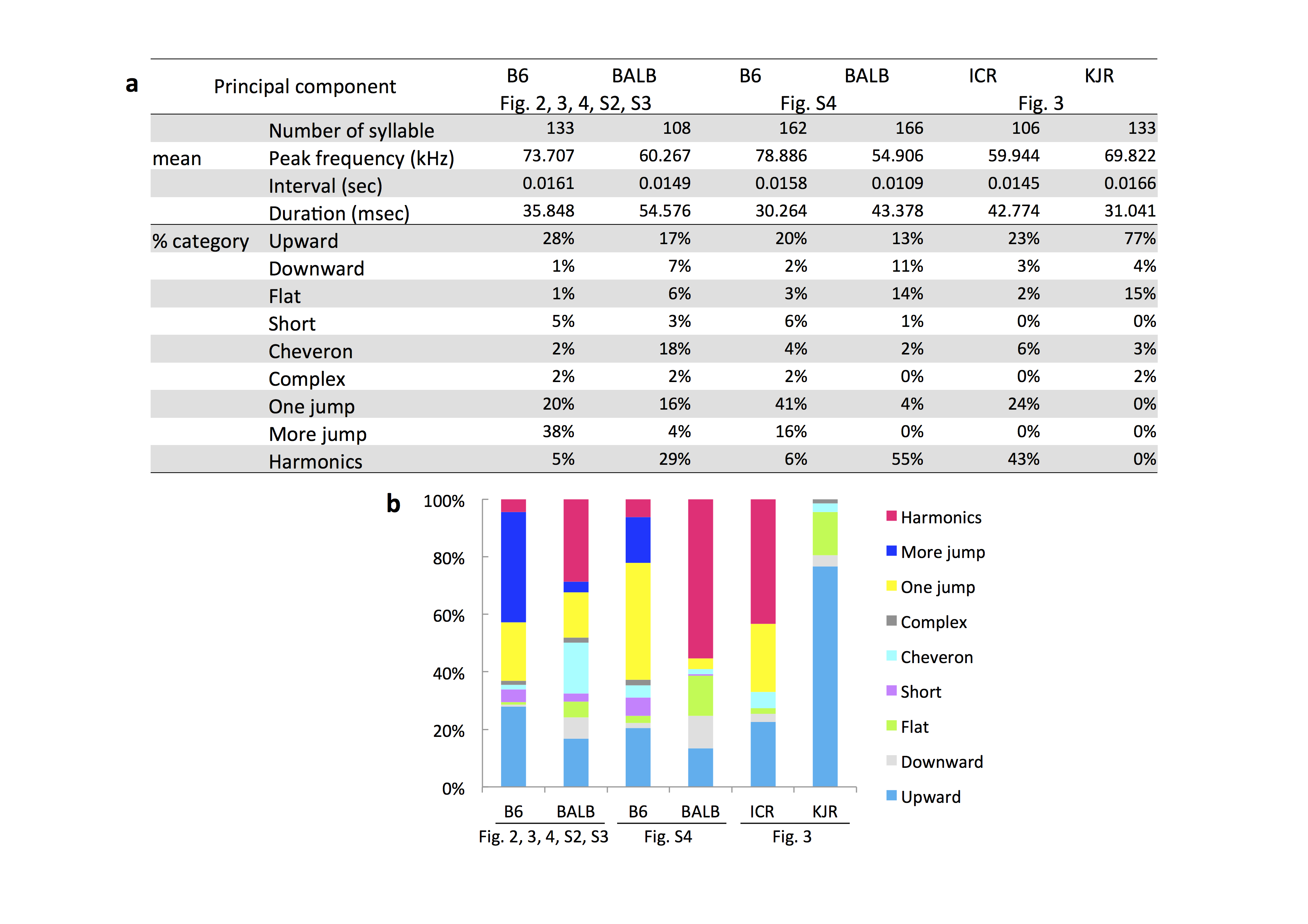

Supplement: Figure S5 — Information about male songs used for each playback test. (a) Principal components in 6 songs used for each playback test. (b) Mean percentage composition of each waveform category in the 6 playback songs. (TIFF) [file pone.0087186.s007.tif]
